# Defect-Adapted Guided Bone Regeneration of the Atrophic Edentulous Maxilla Using a PRGF-Enriched Autogenous Bone/ABBM Composite: A Prospective One-Year Case Series

**DOI:** 10.3390/ma19173640

**Published:** 2026-08-27

**Authors:** Liliana Andrea Faria da Silva, Maria Helena Fernandes, Pedro Sousa Gomes

**Affiliations:** 1 Cirurgia Oral, Faculdade de Medicina Dentária, Universidade do Porto, Rua Dr. Manuel Pereira da Silva, 4200-393 Porto, Portugal; 2 BoneLab, Faculdade de Medicina Dentária, Universidade do Porto, Rua Dr. Manuel Pereira da Silva, 4200-393 Porto, Portugal; mhfernandes@fmd.up.pt; 3 LAQV/REQUIMTE, Faculdade de Medicina Dentária, Universidade do Porto, Rua Dr. Manuel Pereira da Silva, 4200-393 Porto, Portugal

**Keywords:** alveolar ridge augmentation, bone regeneration, maxilla, sinus floor augmentation, plasma rich in growth factors, anorganic bovine bone mineral, cone-beam computed tomography

## Abstract

**Objectives**: We sought to evaluate CBCT-based dimensional outcomes, short-term dimensional changes, the feasibility of delayed implant placement, and complication profiles after defect-adapted guided bone regeneration of the atrophic edentulous maxilla using a PRGF-enriched autogenous bone/anorganic bovine bone mineral composite, including cases requiring simultaneous lateral-window sinus floor elevation. **Materials and Methods**: In this prospective, single-arm, non-randomized clinical case series, 11 patients with an atrophic edentulous maxilla requiring prosthetically guided reconstruction before implant placement were consecutively treated. Eligibility criteria included a residual ridge width < 6 mm and/or residual bone height < 10 mm, with ASA I–II status. Patients underwent defect-adapted GBR with a 60:40 mixture of autogenous bone and anorganic bovine bone mineral combined with plasma rich in growth factors (PRGF), stabilized within a defect-adapted barrier membrane system. Eight patients received combined vertical and horizontal augmentation, and three received horizontal augmentation, with simultaneous sinus floor elevation performed where anatomically indicated. Implants were placed in a delayed protocol at re-entry (6–9 months). Bone height and width were measured using standardized cone-beam computed tomography at baseline, immediately post-operatively, at 6–9 months, and at 12 months at three maxillary reference sites. Selected bone core biopsies obtained at re-entry were processed for descriptive histological evaluation. **Results**: Mean vertical bone dimension increased from 6.8 mm pre-surgically to 14.9 mm immediately postoperatively and measured 14.5 mm at 6–9 months and 13.9 mm at 12 months; mean horizontal bone dimension increased from 4.1 to 8.8 mm and measured 8.6 and 8.2 mm, respectively, at the corresponding follow-up time points. At 12 months, the retained mean dimensional gain was 7.1 mm vertically and 4.1 mm horizontally. Descriptive histological evaluation of selected bone core biopsies showed newly formed bone associated with residual bone-substitute particles and bone–biomaterial interface remodeling. In total, 75 implants were placed according to a delayed protocol. No intra-operative complications occurred. Three low-grade infections resolved without loss of the regenerative construct, whereas one high-grade infection caused complete graft loss at the affected site; it was excluded from the bone-gain analysis. **Conclusions**: Within its limitations, the PRGF-enriched autogenous bone/ABBM composite stabilized within a defect-adapted GBR compartment was associated with substantial vertical and horizontal augmentation of the severely atrophic edentulous maxilla, with limited mean linear dimensional reduction between the immediate postoperative and 12-month assessments. The approach enabled delayed implant placement in prosthetically guided positions. Controlled studies with longer follow-up are required to clarify the contribution of PRGF and validate the long-term functional performance of this multi-component regenerative biomaterial strategy.

## 1. Introduction

Fixed implant-supported rehabilitation is a predictable and widely accepted treatment approach for restoring fully edentulous patients, but its long-term success depends on the availability of adequate three-dimensional bone volume in a prosthetically appropriate position [1,2]. After tooth loss, the alveolar process undergoes progressive and largely irreversible resorption, characterized by marked horizontal contraction and clinically relevant vertical loss during early healing [3,4]. In the maxilla, this process is further compounded by centripetal resorption and sinus pneumatization, progressively reducing both ridge height and width [5]. Consequently, reconstruction of the atrophic edentulous maxilla remains one of the most demanding scenarios in implant dentistry and maxillofacial rehabilitation, requiring not only surgical expertise but also regenerative biomaterial strategies capable of restoring adequate volume and supporting long-term implant stability [6]. In routine clinical practice, these reconstructions are rarely uniform: defect morphology, residual bone anatomy, sinus anatomy, and soft-tissue conditions vary substantially between patients and across maxillary regions.

Several reconstructive approaches have been proposed for the rehabilitation of severely atrophic maxillae, including autogenous onlay block grafting, guided bone regeneration (GBR), ridge splitting, distraction osteogenesis, and sinus floor elevation [6]. Autogenous block grafting has historically been considered a benchmark approach for the atrophic maxilla, but in extensive defects the intra-oral donor supply is often insufficient, while extra-oral harvesting increases morbidity and block grafts may undergo unpredictable resorption [7]. Accordingly, particulate grafting strategies frequently combine autogenous bone with osteoconductive bone substitutes to reduce donor-site requirements while preserving key biological and structural functions within a more adaptable grafting approach [8,9]. In these composite grafts, autogenous bone contributes a biologically active component that supports osteogenesis and remodeling, whereas anorganic bovine bone mineral (ABBM) is usually used to provide a slowly resorbing osteoconductive scaffold that supports bone ingrowth and helps preserve the augmented contour during graft remodeling [9].

To further functionalize particulate graft composites, autologous platelet concentrates have been proposed as biological adjuncts capable of supporting tissue repair, graft cohesion, and early regenerative events; however, these preparations are heterogeneous and differ in cellular composition, fibrin architecture, and growth-factor release profiles [8,10]. Plasma rich in growth factors (PRGF) is an autologous, leucocyte-free platelet preparation belonging to the pure platelet-rich plasma family that delivers platelet-derived mediators within a fibrin scaffold and has been shown to support osteoblast proliferation and migration, and the release of proangiogenic mediators [10,11]. When incorporated into regenerative protocols, PRGF may therefore act as a fibrin-based bioactive adjunct that improves particle cohesion, handling characteristics, and early clot stability [10,11]. Despite this biological and handling rationale, clinical evidence on PRGF-enriched particulate composite grafts for extensive three-dimensional reconstruction of the atrophic maxilla remains limited, particularly when applied across anatomically heterogeneous clinical defects requiring defect-adapted vertical, horizontal, and sinus-floor augmentation strategies.

For a particulate regenerative composite to perform clinically, its biological and scaffold components must be stabilized within a protected, mechanically competent compartment that preserves graft volume during healing. Guided bone regeneration (GBR) provides this barrier-mediated environment, in which the composite graft, blood clot or fibrin matrix, and membrane system act together to support osteoconduction, early biological signaling, mechanical stability, and protection from soft-tissue invasion. This concept is reflected in the PASS principles—primary wound closure, angiogenesis, space maintenance, and clot stability—which summarize the biological and mechanical requirements for predictable bone regeneration [12]. Among these requirements, space maintenance is particularly important in vertical and combined vertical–horizontal defects, where the regenerative compartment must resist soft-tissue compression and preserve the augmented contour throughout healing [9,13,14]. Accordingly, membrane selection should match defect morphology and mechanical demand: titanium-reinforced non-resorbable membranes are typically used when rigid space maintenance is required, whereas collagen membranes may be suitable for more contained horizontal defects with lower intrinsic space-making requirements [15].

In the posterior maxilla, reconstruction is further complicated by sinus pneumatization and reduced residual alveolar height. Lateral-window sinus floor elevation is among the most predictable pre-prosthetic procedures [16,17], with high long-term implant survival when enhanced-surface implants and antrostomy coverage are used [18,19]. However, when posterior maxillary atrophy coexists with horizontal ridge deficiency, sinus floor elevation becomes part of a broader three-dimensional reconstructive strategy rather than an isolated subantral procedure. Under these conditions, the regenerative composite must support both subantral space maintenance and external ridge-contour reconstruction, making posterior maxillary defects a clinically relevant setting for assessing biomaterial performance under routine clinical conditions.

Based on this rationale, the present study aimed to characterize the clinical performance of a PRGF-enriched autogenous bone/ABBM composite stabilized within a defect-adapted GBR compartment for reconstruction of the atrophic edentulous maxilla. Specifically, we assessed CBCT-based vertical and horizontal dimensional changes from baseline through 12 months, delayed implant placement, and the complication profile across anatomically heterogeneous reconstructive scenarios, including cases requiring simultaneous sinus-floor elevation. Selected bone-core biopsies obtained at re-entry were also evaluated descriptively to provide histological context for the regenerated tissue.

## 2. Material and Methods

### 2.1. Study Design, Ethics, and Registration

This was a prospective, single-arm, non-randomized clinical case series evaluating vertical and/or horizontal alveolar ridge augmentation of the edentulous maxilla using a standardized GBR protocol based on a PRGF-enriched autogenous bone/ABBM composite, including simultaneous lateral-window sinus floor elevation where indicated. The study was designed to assess the clinical performance of this multi-component regenerative biomaterial strategy for three-dimensional maxillary reconstruction prior to implant placement. The study was approved by the institutional Ethics Committee for Health (ref. 5/2022) and conducted in accordance with the Declaration of Helsinki. The study was registered at ClinicalTrials.gov (Identifier: NCT07654218). Patients were consecutively enrolled between December 2024 and January 2026. All participants provided written informed consent before enrolment, including consent for the use of anonymized clinical and radiographic data. All surgical procedures were performed by a single experienced surgeon, and all radiographic measurements were performed according to a standardized CBCT-based protocol by a calibrated examiner.

### 2.2. Participants and Eligibility

**Figure 1:**
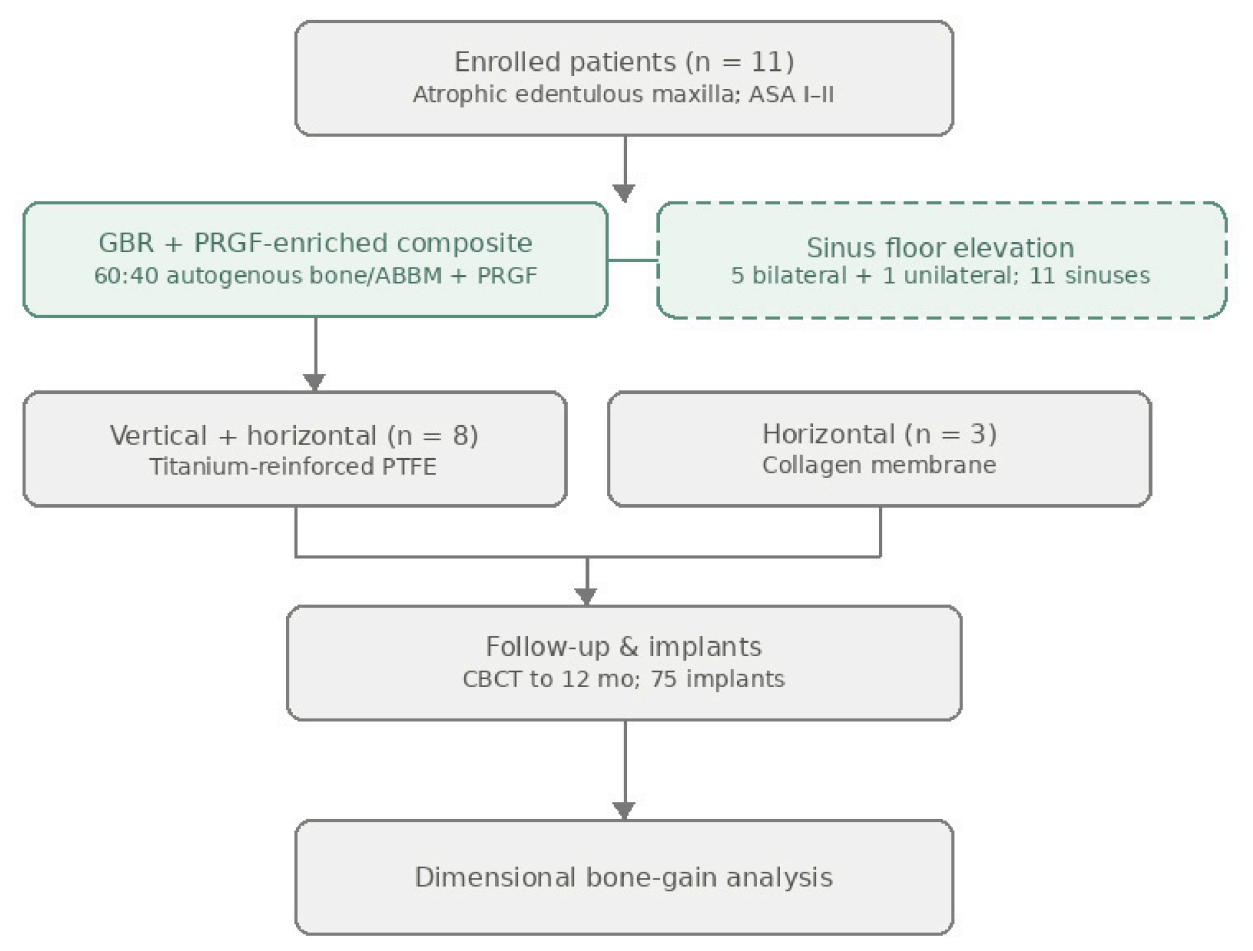
Study workflow. Flow diagram summarizing patient enrolment, defect-adapted treatment allocation, use of the PRGF-enriched autogenous bone/ABBM composite, sinus floor elevation where indicated, CBCT follow-up, delayed implant placement, and dimensional bone-gain analysis.

Patients with an edentulous, atrophic maxilla requiring three-dimensional augmentation prior to implant placement were consecutively enrolled. Eligible participants were adults requiring maxillary reconstruction for prosthetically guided implant-supported rehabilitation. Inclusion criteria were American Society of Anesthesiologists (ASA) physical status I–II and insufficient residual bone volume for implant placement, defined by residual alveolar ridge width below 6 mm and/or residual bone height below 10 mm according to the planned reconstructive indication. Patients were excluded in the presence of general contraindications to oral surgery or implant therapy, uncontrolled systemic disease, active infection at the surgical site, untreated periodontal or mucosal disease, previous radiotherapy to the head and neck region, pregnancy or breastfeeding, heavy smoking, alcohol or substance abuse, or inability to comply with the surgical and follow-up protocol. Each patient was assigned to a defect-adapted reconstructive approach, including combined vertical–horizontal or horizontal augmentation, with simultaneous sinus floor elevation where indicated, according to defect morphology, residual bone anatomy, and sinus pneumatization. Because treatment assignment was determined by defect morphology and reconstructive need, no randomization was performed. The overall patient flow, treatment assignment, and analysis are summarized in Figure 1. 

### 2.3. Surgical Protocol

After a crestal incision and full-thickness flap elevation, the recipient bed was decorticated under copious irrigation to promote bleeding and angiogenesis. Flap design and release were performed to ensure adequate access to the atrophic ridge and allow passive, tension-free primary closure after graft placement. Autogenous bone was harvested intra-orally with a bone scraper and combined with ABBM (Bio-Oss, Geistlich Pharma, Wolhusen, Switzerland) in a 60:40 ratio. 

PRGF was prepared immediately before grafting using the Endoret^®^/PRGF^®^ system (BTI Biotechnology Institute, Vitoria-Gasteiz, Spain). Peripheral venous blood (36 mL per patient) was collected, yielding approximately 18 mL of PRGF. After centrifugation, the plasma fraction located above the leukocyte-rich layer was carefully collected, avoiding aspiration of the buffy coat, in accordance with the leucocyte-free PRGF preparation concept. The selected PRGF fraction was activated with calcium chloride to induce fibrin polymerization and formation of a growth-factor–enriched autologous fibrin matrix. The activated PRGF was then incorporated into the particulate autogenous bone/ABBM mixture immediately before placement, generating a cohesive and moldable composite graft. Because the required graft volume varied according to defect size, a fixed volumetric PRGF-to-graft ratio was not used. The grafted compartment was contained by a defect-adapted barrier membrane system: titanium-reinforced PTFE membranes were used for defects requiring rigid vertical space maintenance and were adapted and stabilized with titanium pins, whereas collagen membranes were used for horizontal augmentations in which rigid vertical support was not required. Where posterior residual bone height was insufficient, lateral-window sinus floor elevation was performed in the same stage, with elevation of the Schneiderian membrane, grafting of the subantral compartment, and coverage of the antrostomy [20]. When sinus floor elevation was combined with ridge augmentation, grafting was planned as part of the same three-dimensional reconstructive strategy, aiming to maintain both the subantral regenerative space and the external ridge contour for subsequent prosthetically guided implant placement. Tension-free primary closure was obtained in all cases. 

Post-operative management included systemic medication, antiseptic mouth rinsing, local wound-care instructions, and scheduled clinical follow-up to monitor healing and detect complications. Postoperative systemic medication consisted of oral amoxicillin 1 g twice daily for 8 days and oral ibuprofen 600 mg twice daily for 5 days. No adjunctive corticosteroids were administered. Patients were instructed to avoid mechanical trauma, prosthetic pressure, and functional loading over the augmented region during the early healing period.

**Figure 2:**
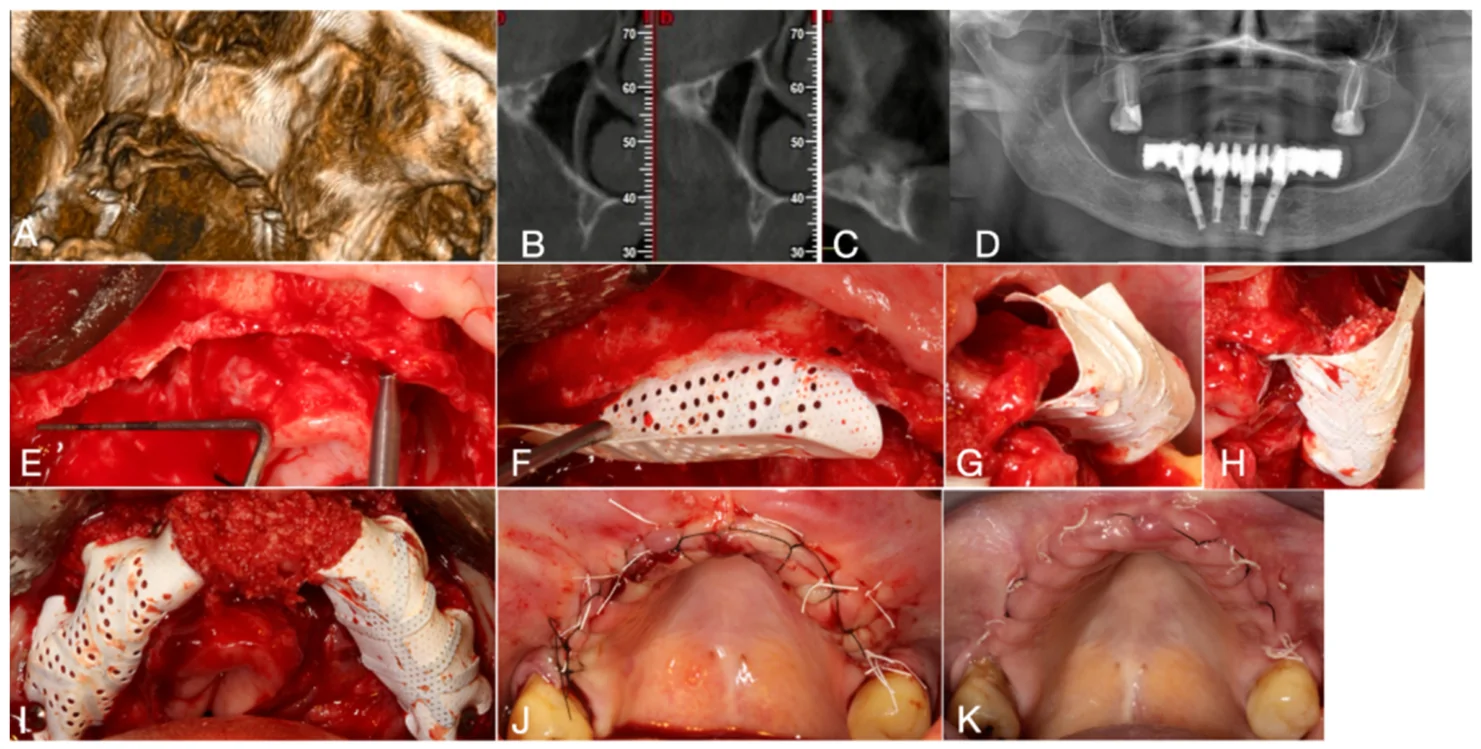
Representative case of reconstruction of the severely atrophic edentulous maxilla by defect-adapted guided bone regeneration combined with plasma rich in growth factors (PRGF) (**A**–**K**). Preoperative three-dimensional CBCT reconstruction (**A**), preoperative CBCT cross-sectional views (**B**,**C**), and preoperative panoramic radiograph (**D**). Clinical photographs show surgical exposure and decortication of the atrophic edentulous ridge (**E**), shaping and palatal stabilization of the titanium-reinforced membrane (**F**), adaptation of the membrane to the three-dimensional contour of the defect (**G**,**H**), placement of the composite graft—a 60:40 mixture of autogenous bone and anorganic bovine bone mineral enriched with PRGF—beneath the pin-stabilized barrier with a collagen overlay (**I**), and tension-free primary closure and early healing (**J**,**K**).

Implants were placed according to a delayed protocol at re-entry, 6–9 months after augmentation. At this stage, the augmented ridge was surgically exposed, non-resorbable barriers and fixation pins were removed where applicable, and the regenerated bone volume was clinically assessed before implant osteotomy preparation. Implants were inserted in prosthetically guided positions within the regenerated bone envelope. No prosthetic functional loading was performed during the observation period. A representative clinical case illustrating the preoperative condition and the main surgical steps of the defect-adapted GBR protocol is shown in Figure 2.

### 2.4. CBCT Acquisition and Radiographic Measurements

Cone-beam computed tomography (CBCT) was obtained pre-surgically (PS), immediately post-operatively (IM), at 6–9 months (PS6–9, coinciding with re-entry) and at 12 months (PS12). All CBCT examinations were acquired using a NewTom Giano^®^ unit (Verona, Italy) with a standardized maxillary imaging protocol, 0.3 mm voxel size, and the smallest field of view sufficient to include the entire edentulous maxilla, maxillary sinuses, and predefined anatomical reference sites. DICOM datasets were imported into DTX Studio Implant^®^ software (Nobel Biocare AB, Gothenburg, Sweden) for standardized radiographic assessment and implant planning. 

**Figure 3:**
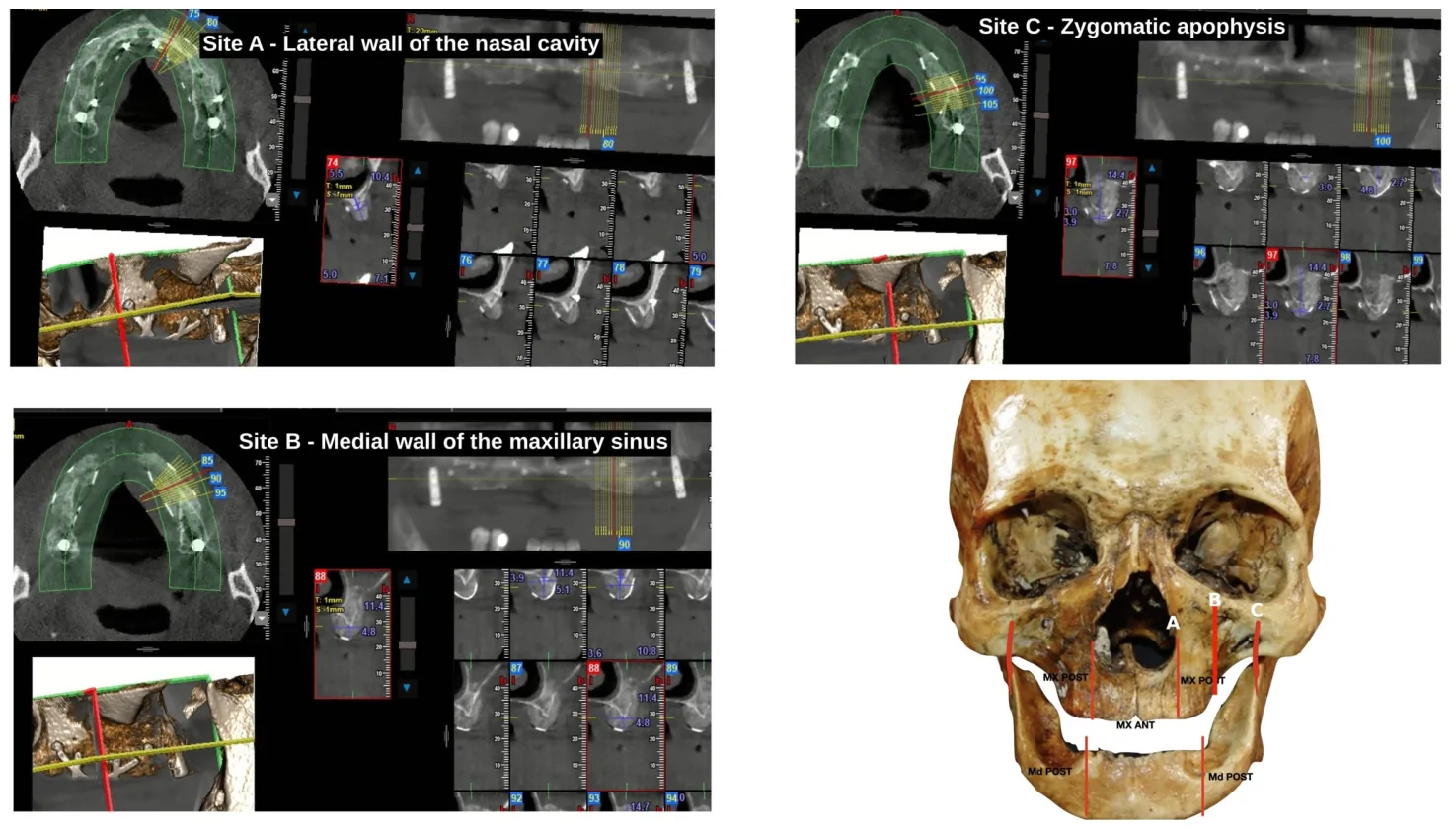
Standardized CBCT measurement reference sites of the maxilla: Site A corresponds to the lateral wall of the nasal cavity in the anterior region; Site B to the medial wall of the maxillary sinus in the middle region; and Site C to the zygomatic apophysis in the posterior region. The skull schematic illustrates the antero-posterior distribution of the three reference sites.

Horizontal and vertical linear measurements were performed on cross-sectional CBCT images at three reproducible maxillary reference sites: the lateral wall of the nasal cavity (Site A, anterior region), the medial wall of the maxillary sinus (Site B, middle region), and the zygomatic apophysis (Site C, posterior region) (Figure 3). These reference sites were selected to provide anterior, middle, and posterior measurements of the reconstructed maxilla. For each patient, corresponding cross-sectional images were identified at each time point using the same anatomical reference site and orientation. 

Vertical bone dimension was measured on each cross-section from the relevant basal anatomical reference, such as the nasal floor or sinus floor region according to site location, to the most coronal aspect of the residual or regenerated ridge. Horizontal bone dimension was measured perpendicular to the ridge axis at the corresponding reference level, using the same orientation and anatomical site across time points. Measurements were therefore intended to reflect site-specific changes in the available bone envelope for prosthetically guided implant placement rather than whole-graft volumetric change. To assess intra-examiner reproducibility, a randomly selected subset of 30 linear measurements was repeated after 2 weeks by the same calibrated examiner. Agreement between the initial and repeated measurements was evaluated using the intraclass correlation coefficient (ICC). No second examiner was involved.

### 2.5. Histological Assessment

Eight bone core biopsies were obtained from regenerated sites in three of the 11 participants using a trephine drill during implant-site preparation, when clinically feasible, and were processed for descriptive histological evaluation. Specimens were fixed in 4% formaldehyde, decalcified in EDTA, embedded in paraffin, sectioned, and stained with haematoxylin and eosin (H&E) for light-microscopic assessment of newly formed bone, residual bone-substitute particles, and the bone–biomaterial interface.

### 2.6. Outcomes and Statistical Analysis

Bone gain was expressed in millimeters and as a percentage of the pre-surgical dimension. Linear dimensional reduction was expressed in millimeters and as a percentage of the immediate postoperative dimension, calculated from the corresponding mean dimensional values. The primary outcomes were CBCT-based vertical and horizontal dimensional changes of the augmented maxilla between PS and each post-operative time point. Dimensional changes were assessed by comparing the immediate post-operative measurements with the 6–9-month and 12-month measurements. Secondary outcomes included the number of implants placed and the incidence, severity, and management of surgical or healing complications.

Complications were prospectively monitored throughout the surgical, healing, re-entry, and follow-up periods. Low-grade complications were defined as events managed conservatively without loss of the regenerative construct, whereas high-grade complications were defined as events requiring additional surgical management and/or resulting in partial or complete graft loss.

Continuous variables were summarized using descriptive statistics, including mean, standard deviation, and range where applicable. Categorical variables were presented as absolute and relative frequencies. Given the prospective, single-arm case-series design, the modest sample size, and the absence of a control group, no inferential statistical testing was performed. The measurement site corresponding to the area with high-grade infection and graft loss was excluded from the bone-gain analysis, while unaffected sites from the same patient were retained.

Histological findings were considered descriptive and were not included in the primary dimensional outcome analysis.

## 3. Results

### 3.1. Study Population and Treatment Distribution

Eleven patients were included in the study, comprising 9 women and 2 men, with a mean age of 52.5 years (range 40–67; SD 9.9). Six patients presented a thin soft-tissue phenotype and five presented a thick phenotype. According to defect morphology and reconstructive need, eight patients underwent combined vertical–horizontal augmentation and three underwent horizontal augmentation. Sinus floor elevation was performed in 6 of 11 patients, corresponding to 11 elevated sinuses: five bilateral and one unilateral sinus augmentation procedures. Patient-level demographic, anatomical, reconstructive, complication-related, and implant-placement data are summarized in Table 1.

A total of 75 implants were placed according to a delayed protocol after the graft-healing period. A representative case illustrating the preoperative condition and surgical reconstruction with the PRGF-enriched autogenous bone/ABBM composite is shown in Figure 2.

### 3.2. CBCT Measurement Reproducibility

Repeated CBCT measurements showed excellent intra-examiner reproducibility, with an intraclass correlation coefficient (ICC) of 0.93 (95% CI: 0.86–0.97), supporting the consistency of the standardized linear measurement protocol.

### 3.3. CBCT-Based Dimensional Outcomes

CBCT-based linear measurements showed substantial vertical and horizontal dimensional gains after reconstruction, with most of the augmented dimensions maintained throughout follow-up (Table 2). Mean vertical bone dimension increased from 6.8 mm pre-surgically to 14.9 mm immediately post-operatively, corresponding to an immediate gain of 8.1 mm, or 119.1% relative to baseline. Mean vertical dimension measured 14.5 mm at 6–9 months and 13.9 mm at 12 months, corresponding to a retained 12-month gain of 7.1 mm, or 104.4% relative to baseline.

Mean horizontal bone dimension increased from 4.1 mm pre-surgically to 8.8 mm immediately post-operatively, corresponding to an immediate gain of 4.7 mm, or 114.6% relative to baseline. Horizontal dimension was maintained at 8.6 mm at 6–9 months and 8.2 mm at 12 months, corresponding to a retained 12-month gain of 4.1 mm, or 100.0% relative to baseline.

Between the immediate post-operative and 12-month measurements, mean linear dimensional reduction was 1.0 mm (6.7%) vertically and 0.6 mm (6.8%) horizontally. A complementary subgroup analysis was performed using the patient-level maximum vertical-gain measurements at Site C. Patients undergoing sinus floor elevation (n = 6) demonstrated mean gains of 15.7 ± 3.3 mm at both 9 and 12 months, whereas patients without sinus elevation and with evaluable vertical-gain measurements (n = 2) demonstrated gains of 6.0 ± 1.3 mm and 6.0 ± 1.2 mm, respectively (Table 3). These findings provide descriptive evidence that the greater posterior vertical gain was strongly influenced by simultaneous sinus floor elevation. This analysis represents maximum vertical gain and is distinct from the absolute longitudinal Site C dimensions reported in Table 2.

**Figure 4:**
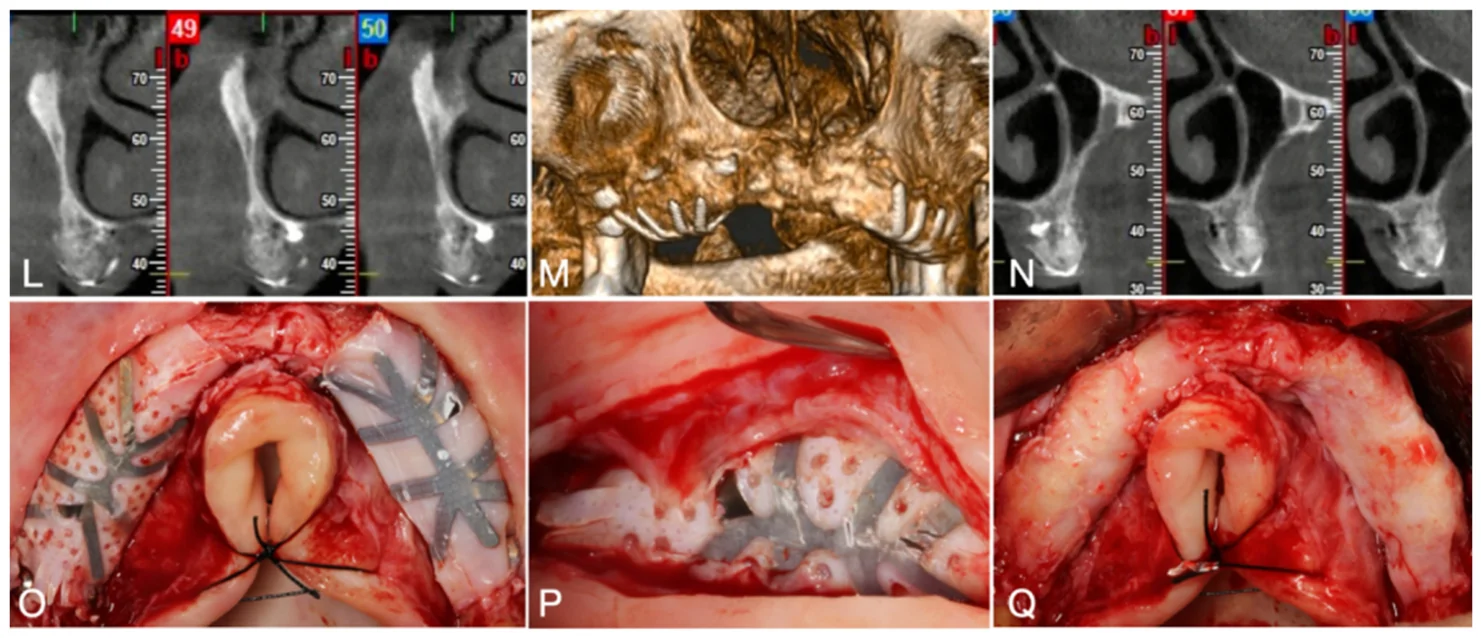
Representative follow-up and re-entry findings after defect-adapted guided bone regeneration of the severely atrophic edentulous maxilla (**L**–**Q**). CBCT cross-sectional views at re-entry (**L**,**N**) and three-dimensional CBCT reconstruction at follow-up (**M**) illustrate the regenerated bone envelope. Clinical views at re-entry show the barrier and titanium pins in situ (**O**,**P**) and the regenerated ridge after barrier removal (**Q**).

Representative CBCT follow-up and re-entry views illustrating the regenerated bone envelope are shown in Figure 4.

### 3.4. Complications and Implant Placement

No intra-operative complications were recorded. Three low-grade infections occurred during healing (3/11 participants; 27.3%) and resolved with conservative management, without loss of the regenerative construct or compromise of subsequent implant placement. One delayed high-grade infection occurred in one participant (1/11; 9.1%) and resulted in complete graft loss at the affected site; this site was excluded from the bone-gain analysis, while unaffected sites from the same patient were retained. Of the 75 implants placed according to the delayed protocol, none failed to integrate or was lost before the 12-month endpoint, corresponding to an implant retention rate of 100% (75/75). No implants were functionally loaded during the observation period. 

**Figure 5:**
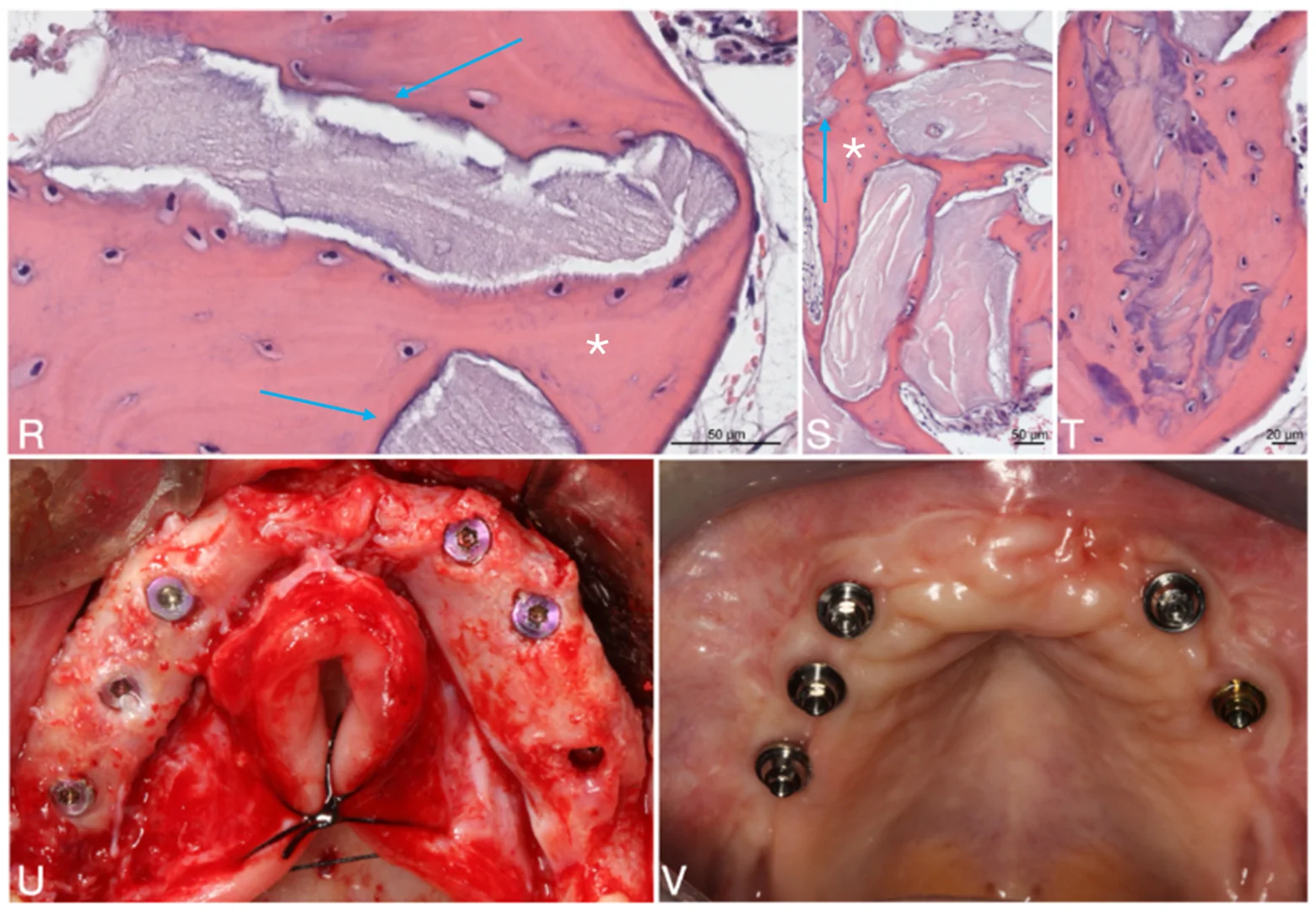
Representative histological and implant-stage findings after defect-adapted guided bone regeneration of the severely atrophic edentulous maxilla (**R**–**V**). Histological sections stained with haematoxylin–eosin show residual bone-substitute particles bridged and surrounded by newly formed bone with osteocytes in lacunae (**R**,**S**), and a higher-magnification view of the bone–biomaterial interface showing a reversal line and active remodelling (**T**). Scale bars: (**R**,**S**) 50 µm; (**T**) 20 µm. Clinical views show the regenerated ridge with implants in place (**U**) and the occlusal view with abutments in situ (**V**). White asterisks indicate areas of newly formed bone bridging residual graft particles, whereas blue arrows indicate remodeling interfaces between newly formed bone and the residual graft particles.

Histological evaluation of bone-core biopsies obtained from selected participants showed newly formed bone associated with residual bone-substitute particles and evidence of bone–biomaterial interface remodeling. Representative histological and implant-stage views are shown in Figure 5.

## 4. Discussion

This prospective case series evaluated the clinical performance of a PRGF-enriched autogenous bone/ABBM composite stabilized within a defect-adapted GBR compartment for reconstruction of the atrophic edentulous maxilla. Across anatomically heterogeneous defects, this composite-based regenerative approach was associated with substantial vertical and horizontal dimensional gains, limited short-term dimensional reduction, and feasibility of delayed implant placement.

The dimensional maintenance observed in this series is consistent with the complementary functions of the regenerative construct. Autogenous bone provides a biologically active component [21,22], ABBM contributes a slowly resorbing osteoconductive scaffold for contour stability [23], PRGF adds a fibrin-mediated phase that may improve graft cohesion and early regenerative events [11], and the membrane–fixation system provides barrier protection and mechanical stabilization [9,24]. Thus, the protocol should be interpreted as an integrated biomaterial strategy in which biological activity, scaffold persistence, fibrin-mediated cohesion, and barrier-supported space maintenance act together to support regeneration.

The magnitude and maintenance of the dimensional gains are clinically relevant, particularly because reconstruction of the atrophic edentulous maxilla must provide sufficient bone volume for prosthetically guided implant placement [25]. In the present series, both vertical and horizontal dimensions increased substantially after surgery and remained largely preserved at 12 months, indicating that most of the surgically created bone envelope was maintained during early healing (Table 2). This dimensional maintenance is important not only for implant placement, but also for re-establishing a prosthetically appropriate three-dimensional corridor in the severely resorbed maxilla. The limited linear dimensional reduction observed between the immediate post-operative and 12-month measurements is consistent with controlled remodeling of particulate grafted sites rather than collapse of the augmented contour [26]. Descriptive histological evaluation of selected bone core biopsies provided complementary tissue-level context, showing newly formed bone associated with residual bone-substitute particles and features of bone–biomaterial interface remodeling (Figure 5).

Published GBR outcomes provide useful, although indirect, benchmarks for interpreting the present findings. A systematic review reported a weighted mean vertical gain of 4.18 mm for GBR procedures [13], while studies using titanium-reinforced PTFE barriers combined with autogenous bone and ABBM reported mean vertical gains of 5.45 mm and 5.2 ± 2.4 mm [9,24]. A retrospective study using particulate autogenous and xenogeneic grafts combined with platelet concentrate reported gains of 5.6 ± 2.6 mm vertically and 5.9 ± 2.4 mm horizontally [8]. In the present series, the retained changes at 12 months were 7.1 mm vertically and 4.1 mm horizontally. However, direct comparison is limited by differences in defect morphology, measurement methodology, anatomical reference sites, follow-up intervals, and regenerative protocols. In particular, the posterior vertical measurements in the present study were influenced by simultaneous sinus floor elevation. These published outcomes therefore provide descriptive context for the magnitude of the observed changes but should not be interpreted as demonstrating equivalence or superiority.

Vertical and horizontal reconstruction should be interpreted in relation to their different mechanical requirements. Vertical and combined vertical–horizontal defects require a regenerative compartment capable of resisting soft-tissue compression and maintaining the augmented contour throughout healing; this supports the use of titanium-reinforced non-resorbable barriers when rigid space maintenance is required [9,24]. By contrast, contained horizontal defects generally impose lower space-making demands and may be managed with collagen membranes when sufficient defect morphology and graft stability are present [15]. In this series, preservation of both vertical and horizontal dimensions supports the rationale for defect-adapted barrier selection.

The posterior outcomes should also be interpreted considering the simultaneous sinus floor elevation performed where indicated. The greater absolute vertical gain observed at the posterior reference site is consistent with the additional subantral volume created by lateral-window sinus augmentation, rather than reflecting crestal GBR alone [27]. Importantly, in these cases, sinus elevation was integrated into the same reconstructive strategy used to restore the external ridge contour, so the material construct had to support both subantral space maintenance and lateral ridge reconstruction. The maintenance of the posterior vertical dimension during follow-up suggests that this combined approach contributed to preserving the surgically created posterior bone envelope.

The complication profile should be interpreted in the context of the anatomical complexity and extent of the reconstructions performed. No intra-operative complications were recorded, and most healing events were manageable without loss of the regenerative construct. Three low-grade infections resolved with conservative treatment, whereas one delayed high-grade infection resulted in complete graft loss at the affected site. Although this event underscores the biological and technical sensitivity of extensive maxillary augmentation, the overall reconstructive pathway remained clinically feasible, with delayed placement of 75 implants after graft healing. These findings reinforce the importance of careful soft-tissue management, passive primary closure, membrane stability, and close post-operative monitoring in vertical and combined vertical–horizontal GBR procedures [28]. 

This study has several limitations that should be considered when interpreting the findings. First, the single-arm design, modest sample size, anatomical heterogeneity, and absence of a comparator group mean that the findings should be interpreted as evidence of clinical performance rather than comparative efficacy. Although anatomical heterogeneity limits standardization, it also reflects routine oral-surgical bone regeneration, in which reconstructive protocols must be adapted to patient-specific defect morphology, residual bone anatomy, sinus anatomy, soft-tissue conditions, and prosthetic requirements. From a translational perspective, evaluation across such heterogeneous clinical scenarios may complement conventional clinical trials by capturing treatment performance in routine clinical populations and complex care settings [29]. Second, because the regenerative protocol combined autogenous bone, ABBM, PRGF, barrier membranes, and defect-adapted fixation, the independent contribution of each individual component cannot be isolated. Third, histological assessment was limited to the descriptive evaluation of eight bone-core biopsies obtained from three participants. Quantitative histomorphometry, including the percentages of newly formed bone and residual graft particles, was not performed; consequently, the histological findings should be interpreted as illustrative and cannot be considered representative of the entire cohort. Finally, dimensional assessment was based on standardized linear CBCT measurements at predefined anatomical reference sites rather than full three-dimensional volumetric analysis, and the follow-up period was limited to early dimensional changes and delayed implant placement, without assessment of long-term prosthetic loading outcomes.

## 5. Conclusions

Within the limitations of this prospective case series, a PRGF-enriched autogenous bone/ABBM composite stabilized within a defect-adapted GBR compartment showed favorable CBCT-based clinical performance for reconstruction of the atrophic edentulous maxilla. The approach was associated with clinically relevant vertical and horizontal dimensional gains, limited short-term dimensional reduction, and delayed implant placement across anatomically heterogeneous reconstructive scenarios. Future controlled studies incorporating volumetric assessment and longer follow-ups after prosthetic loading are needed to validate the long-term performance of this integrated regenerative biomaterial strategy.

## Figures and Tables

**Table 1:** Distribution of demographics, defect type, phenotype, sinus elevation, complications and implants placed (n = 11).

Patient	Age (y)	Sex	Procedure	Defect Type	Phenotype	Sinus Lift	Complications	Implants (n)
I	43	F	V/H GBR	Vertical/Horizontal	Thin	Bilateral	None	8
II	62	F	V/H GBR	Vertical/Horizontal	Thick	Bilateral	None	7
III	46	F	V/H GBR	Vertical/Horizontal	Thick	No	None	7
IV	48	F	V/H GBR	Vertical/Horizontal	Thin	Bilateral	Low-grade infection (left)	8
V	41	M	V/H GBR	Vertical/Horizontal	Thin	Bilateral	Low-grade infection (left)	8
VI	63	F	V/H GBR	Vertical/Horizontal	Thick	No	None	6
VII	40	F	V/H GBR	Vertical/Horizontal	Thick	Bilateral	Low-grade infection (left)	6
VIII	67	M	H GBR	Horizontal	Thick	No	None	6
IX	52	F	H GBR	Horizontal	Thin	No	None	6
X	64	F	Right: V/H GBR Left: H GBR	Vertical/Horizontal	Thin	No	High-grade infection (right); graft loss	7
XI	52	F	H GBR	Horizontal	Thin	Unilateral	None	6
Mean/total	52.5 (40–67); SD 9.9	9 F, 2 M	—	8 vertical/horizontal; 3 horizontal	6 thin; 5 thick	11 sinuses (5 bilateral + 1 unilateral)	3 low-grade; 1 high-grade infection	75 total

Abbreviations: V/H GBR, vertical and horizontal guided bone regeneration using a titanium-reinforced PTFE membrane and composite graft; H GBR, horizontal guided bone regeneration using a collagen membrane and composite graft.

**Table 2:** Linear horizontal and vertical bone dimensions and derived dimensional changes at each follow-up, measured by CBCT at three maxillary reference sites.

Site	PS (mm)	IM (mm)	6–9 mo (mm)	12 mo (mm)	Gain PS–IM, mm (%)	Gain PS–6–9 mo, mm (%)	Gain PS–12 mo, mm (%); Resorption IM–12 mo, mm (%)
Horizontal bone changes (HOR)							
Site A	4.0 ± 1.5 (0.9–7.0)	7.8 ± 1.2 (5.8–10.8)	7.5 ± 1.6 (3.6–10.8)	7.2 ± 1.4 (3.6–9.7)	3.8 (95.0)	3.5 (87.5)	3.2 (80.0); 0.6 (−7.7)
Site B	3.6 ± 1.6 (1.2–7.6)	9.3 ± 1.4 (7.2–12.3)	9.1 ± 1.9 (3.1–12.3)	8.5 ± 1.7 (3.1–11.3)	5.7 (158.3)	5.5 (152.8)	4.9 (136.1); 0.8 (−8.6)
Site C	4.7 ± 2.0 (2.1–9.9)	9.4 ± 2.1 (6.3–13.8)	9.3 ± 2.1 (6.3–13.8)	9.0 ± 2.0 (6.2–13.8)	4.7 (100.0)	4.6 (97.9)	4.3 (91.5); 0.4 (−4.3)
**Total mean**	**4.1**	**8.8**	**8.6**	**8.2**	**4.7 (114.6)**	**4.5 (109.8)**	**4.1 (100.0);** **0.6 (−6.8)**
Vertical bone changes (VER)							
Site A	7.5 ± 3.5 (2.7–13.7)	12.9 ± 2.9 (7.6–17.3)	12.9 ± 3.1 (7.6–17.3)	12.3 ± 3.4 (7.0–17.0)	5.4 (72.0)	5.4 (72.0)	4.8 (64.0); 0.6 (−4.7)
Site B	9.3 ± 3.3 (3.6–14.5)	13.7 ± 2.4 (9.3–17.4)	13.2 ± 2.6 (9.0–17.4)	12.9 ± 2.6 (8.6–17.0)	4.4 (47.3)	3.9 (41.9)	3.6 (38.7); 0.8 (−5.8)
Site C	3.5 ± 1.9 (1.8–7.9)	18.0 ± 7.1 (8.4–26.7)	17.5 ± 6.9 (8.4–26.7)	16.7 ± 6.6 (8.1–26.1)	14.5 (414.3)	14.0 (400.0)	13.2 (377.1); 1.3 (−7.2)
Total mean	6.8	14.9	14.5	13.9	8.1 (119.1)	7.7 (113.2)	7.1 (104.4); 1.0 (−6.7)

Values at PS, IM, 6–9 months, and 12 months are presented as mean ± SD (range). Gain values are presented as the absolute change in millimeters, followed by the percentage change relative to the pre-surgical dimension in parentheses. Dimensional reduction is presented as the absolute reduction in millimeters, followed by the percentage reduction relative to the immediate postoperative dimension in parentheses.

**Table 3:** Descriptive subgroup analysis of maximum vertical bone gain at the posterior reference site (Site C) according to sinus floor elevation status.

Site C Subgroup	n	Vertical Gain, 9 mo (mm)	Vertical Gain, 12 mo (mm)
Sinus floor elevation	6	15.7 ± 3.3	15.7 ± 3.3
No sinus floor elevation	2	6.0 ± 1.3	6.0 ± 1.2

Values are presented as mean ± standard deviation.

## Data Availability

The data presented in this study are available on request from the corresponding author due to privacy reasons.
